# Bergenin Attenuates Hepatic Fibrosis by Regulating Autophagy Mediated by the PPAR-*γ*/TGF-*β* Pathway

**DOI:** 10.1155/2020/6694214

**Published:** 2020-12-31

**Authors:** Yujing Xia, Jingjing Li, Kan Chen, Jiao Feng, Chuanyong Guo

**Affiliations:** Department of Gastroenterology, Shanghai Tenth People's Hospital, School of Clinical Medicine of Nanjing Medical University, Shanghai 200072, China

## Abstract

Liver fibrosis is a pathological process involving diffuse extracellular matrix (ECM) deposition in the liver. It is typical of many chronic liver diseases, including cirrhosis, and effective drugs are needed. In this study, we explored the protective effect of bergenin on liver fibrosis induced by carbon tetrachloride and bile duct ligation. A variety of molecular biological methods (qRT-PCR, western blotting, and immunohistochemistry) were employed to confirm the increased degree of hepatocyte injury and ECM formation in the disease model, consistent with autophagy and activation of the TGF-*β* pathway. Bergenin activated PPAR-*γ* and inhibited TGF-*β* and autophagy and decreased liver fibrosis by inhibiting hepatocyte necrosis and ECM formation in a dose-dependent manner. The results suggest that bergenin may be a promising drug candidate for the treatment of liver fibrosis.

## 1. Introduction

Liver fibrosis is a pathophysiological process in which various pathogenic factors continually damage the liver, resulting in extracellular matrix (ECM) deposition and fibrous scar formation [1]. According to statistics, more than one million people worldwide die of end-stage liver disease caused by liver fibrosis every year [2]. Therefore, in recent years, experts in the field have explored treatments for liver diseases, especially liver fibrosis and cirrhosis. The consensus is that new drugs are needed to improve both diagnosis and treatment.

Peroxisome proliferator-activated receptor (PPAR) is a ligand-activated receptor belonging to the type II nuclear hormone receptor superfamily that includes PPAR-*α*, PPAR-*β*/*δ*, and PPAR-*γ* subtypes [3, 4]. PPAR-*γ* is a key transcription factor of cell differentiation, which is closely related to fibrosis in important organs [5, 6]. Stavniichuk et al. confirmed that dual soluble epoxide hydrolase inhibitors can reduce renal fibrosis by activating PPAR-*γ*, and the same effect was observed in heart and lung fibrosis [7–9]. In liver fibrosis, PPAR-*γ* is involved in hepatic stellate cell (HSC) activation and fibroblast transformation, which can reduce the overexpression of *α*-smooth muscle actin (*α*-SMA), type I collagen, and hydroxyproline in HSCs and thereby inhibit liver fibrosis [10–12].

Autophagy, an important form of programmed cell death, is a highly conserved degradation process mediated by lysosomes in eukaryotes. The autophagy-related gene LC3-II was significantly upregulated in a carbon tetrachloride- (CCl_4_-) induced liver fibrosis model, and inhibition of autophagy activity could delay its progress. This may be because autophagy-mediated lipid degradation provides energy for HSC activation, thereby promoting ECM formation and the progression of liver fibrosis. However, PPAR-*γ* activation has been linked to autophagy [13, 14], but whether PPAR-*γ* plays an important role in the occurrence and development of liver fibrosis requires further investigation.

Existing liver fibrosis drugs acting at a single target are not particularly effective, and they cause unwanted side effects. Some active components of traditional Chinese medicines, such as procyanidin, crocin, astaxanthin, and fucoidan, are reported to exert strong antifibrosis effects [12, 15–17]. Some reportedly regulate adipocytokines, thereby reducing liver inflammation and lowering oxidative stress [17, 18]. Bergenin is a natural secondary metabolite extracted from the roots, bark, and leaves of many families and genera of plants. Its pharmacological activities are diverse, and antitumour, antiviral, immune enhancement, wound repair, anticoagulant, analgesic, antitussive, antifungal, antiarrhythmic, antimalarial, and anti-inflammatory activities have been reported [19–23]. However, liver fibrosis activity has not been reported.

The etiology and mechanism of liver fibrosis are complex. Traditional Chinese medicines can inhibit the activation of HSCs and exert antifibrosis effects via different mechanisms. The aim of the present study was to explore the antifibrosis effect of bergenin and its action mechanism based on the successful establishment of a liver fibrosis model.

## 2. Materials and Methods

### 2.1. Establishment of a Hepatic Fibrosis Model

C57 mice weighing 20-25 g were purchased from Shanghai Experimental Animal Co., Ltd. (Shanghai, China). They were reared at 25°C under a 12 h light/12 h dark cycle. All mice were allowed free access to food and water. The CCl_4_-induced liver fibrosis model was established by intraperitoneal injection of 1 mL/kg body weight CCl_4_ (1 : 10 *v*/*v*; Sigma-Aldrich, St. Louis, MO, USA) in olive oil twice a week for 8 weeks [15]. In the BDL model, C57 mice were fasted for 12 h, injected intraperitoneally with 1.25% Nembutal (Sigma-Aldrich), anesthetised, and disinfected. Skin and muscle were removed layer by layer from the midline of the abdomen and ~1 cm above the perineum. The transparent bile duct accompanying the portal vein was found in the hilar region, and two 6-0 surgical sutures were embedded, and surgical knots were made. After confirming that there was no visceral injury or bleeding in the abdominal cavity, the abdomen was closed layer by layer and disinfected again. Mice were resuscitated in a dry and warm environment [24]. All animal experiments were carried out according to, and approved by, the Animal Care and Use Committee of Nanjing Medical University.

### 2.2. Reagents and Experiment Design

Bergenin (CAS: 477-90-7, purity ≥ 98.0%) was purchased from Sigma-Aldrich and dissolved in physiological saline. Primary antibodies *α*-SMA, CoI-I, TIMP1, LC3-I/II, Beclin-1, and *β*-actin were acquired from Proteintech Group (Chicago, IL, USA), and PPAR-*γ*, RXR-*α*, TGF-*β*1, Smad2, Smad3, and p-Smad2/3 were from Cell Signaling Technology (Danvers, MA, USA). SYBR Premix Ex Taq was purchased from TaKaRa Biotechnology (Dalian, China).

A total of 64 mice were randomly divided into the CCl_4_ model group and the BDL model group, and serum and liver tissue samples were obtained as follows:

*Sham operation group (sham,n* = 8): intragastric administration of normal saline.

*Model group (CCl_4_ or BDL,n* = 8): model established as above.

*Low dose group (CCl_4_/BDL+B20,n* = 8): daily gavage, bergenin (20 mg/kg).

*High dose group (CCl_4_/BDL+B40,n* = 8): daily gavage, bergenin (40 mg/kg).

### 2.3. Assessment of Liver Function

Serum alanine aminotransferase (ALT), aspartate aminotransferase (AST), and hydroxyproline were determined using an Olympus AU1000 Automatic Chemical Analyzer (Olympus Corporation, Tokyo, Japan) in the hospital laboratory.

### 2.4. Pathological Evaluation

Liver tissue was used to prepare paraffin sections that were stained with hematoxylin and eosin (HE) according to the manufacturer's instructions. HE staining solution is alkaline, which stains chromatin and nucleic acid in the nucleus purple/blue, and eosin is an acidic dye, which stains components in the cytoplasm and extracellular matrix red, thereby revealing cell necrosis. In addition, Masson staining was used to probe the degree of fibrosis. Collagen fibers stain blue, and muscle fibers stain red, revealing fibers and inflammatory factors in tissues.

### 2.5. Quantitative Real-Time PCR

Total RNA was extracted from freeze-dried tissue and analysed for purity and concentration. RNA was reverse-transcribed into cDNA and stored at -20°C. Each 20 *μ*L reaction included a predenaturation step at 93°C for 2 min, followed by 40 cycles of 1 min at 93°C, 1 min at 55°C, and 1 min at 72°C, and a final extension at 72°C for 7 min. Expression levels of target genes were determined relative to *β*-actin. The sequences of primers used in the experiment are shown in Table 1.

### 2.6. Western Blotting

Total protein was extracted from tissues, quantified by bicinchoninic acid protein assay (Kaiji, China), mixed with 5x loading buffer, and stored at -20°C. Based on the protein molecular weight, proteins were separated by sodium dodecyl sulphate-polyacrylamide gel electrophoresis (SDS-PAGE) at 80 V using appropriate gels. When the sample reached the lower layer of the gel, the voltage was changed to 120 V, and electrophoresis was stopped when the bromophenol blue indicator reached the bottom of the separating gel. Proteins were transferred to a polyvinylidene fluoride (PVDF) membrane at 200 mA, then incubated on a decolourising shaker at room temperature for 1 h. The PVDF membrane was incubated overnight at 4°C with primary antibodies diluted in phosphate-buffered saline (PBS) containing Tween (PBST). After washing with fresh PBST, the membrane was incubated in the secondary antibody solution for 1 h, then quickly rinsed. An Odyssey Two-colour Infrared Laser Imaging System (LI-COR Biosciences, Lincoln, NE, USA) was used to scan and image the membranes. The quantitative evaluation was determined by relative band density.

### 2.7. Immunohistochemical Staining

The prepared paraffin sections were placed in a 60°C incubator and incubated for 120 min. After a series of dewaxing and hydration treatments, they were incubated at room temperature with 3% H_2_O_2_ for 10 min to eliminate endogenous peroxidase activity. After washing with distilled water to elicit antigen repair, the slices were placed in a container containing PBS and heated in a microwave oven for 15 min to keep the liquid temperature in the container between 92°C and 98°C, then cooled at room temperature for 20 min. After blocking, samples were incubated again at room temperature for 15 min. The working solution of the first antibody was added dropwise overnight at 4°C. After adding the second antibody, samples were washed and stained with DAB. After staining with HE and mixing with hydrochloric acid and alcohol, samples were dehydrated until transparent, sealed with neutral resin and a cover glass, and visualised under a light microscope, revealing the target molecules as yellow particles.

### 2.8. Electron Microscopy

Fresh liver tissues were fixed with 3% glutaraldehyde, incubated with 0.2 mM calcium carbonate buffer for 4 h, and then fixed with 1% osmium tetroxide for 1 h. Samples were dehydrated using a series of ethanol solutions, soaked with epoxy resin, and sliced. Autophagy was observed using a JEM-1230 electron microscope (JEOL, Tokyo, Japan).

### 2.9. Statistical Analysis

SPSS 22.0 software (IBM Corporation, Armonk, NY, USA) was used for statistical analysis. Data were compared as means ± standard deviation calculated by Student-Newman-Keuls tests and one-way analysis of variance (ANOVA). A *p* value < 0.05 was considered statistically significant.

## 3. Results

### 3.1. Bergenin Significantly Decreases Liver Fibrosis

**Figure 1:**
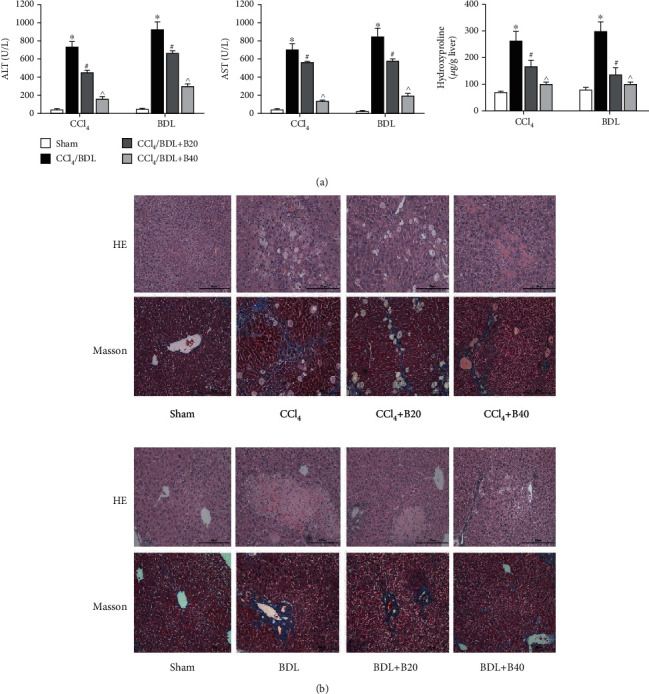
Bergenin significantly decreases liver fibrosis. (a) Levels of serum ALT and AST expressed as the mean ± SD (*n* = 8). (b) HE and Masson staining of liver sections (original magnification = 200x).

Liver enzymes and hydroxyproline are important indicators that reflect the severity of liver fibrosis; hence, serum and pathological examination was carried out to evaluate the effects of the drug. The results showed that levels of ALT and AST in the CCl_4_ and BDL groups were increased, while levels of liver enzymes in drug treatment groups were significantly decreased, and the effect was more obvious with an increasing dose (Figure 1(a)). In accordance with the levels of liver enzymes, hydroxyproline was increased in the disease model groups, while bergenin treatment significantly reduced hydroxyproline levels (Figure 1(a)). HE staining revealed degeneration and necrosis of hepatocytes and proliferation of connective tissue in the disease model groups, and inflammatory cell infiltration was observed in some areas. Additionally, numerous blue collagen fibers were observed by Masson staining. Compared with the disease model groups, necrosis was improved in the drug groups, and the quantity of collagen fibers was decreased in a dose-dependent manner (Figure 1(b)). These results suggest that bergenin can effectively decrease levels of liver enzymes and alleviate liver fibrosis.

### 3.2. Bergenin Inhibits the Formation of Extracellular Matrix

**Figure 2:**
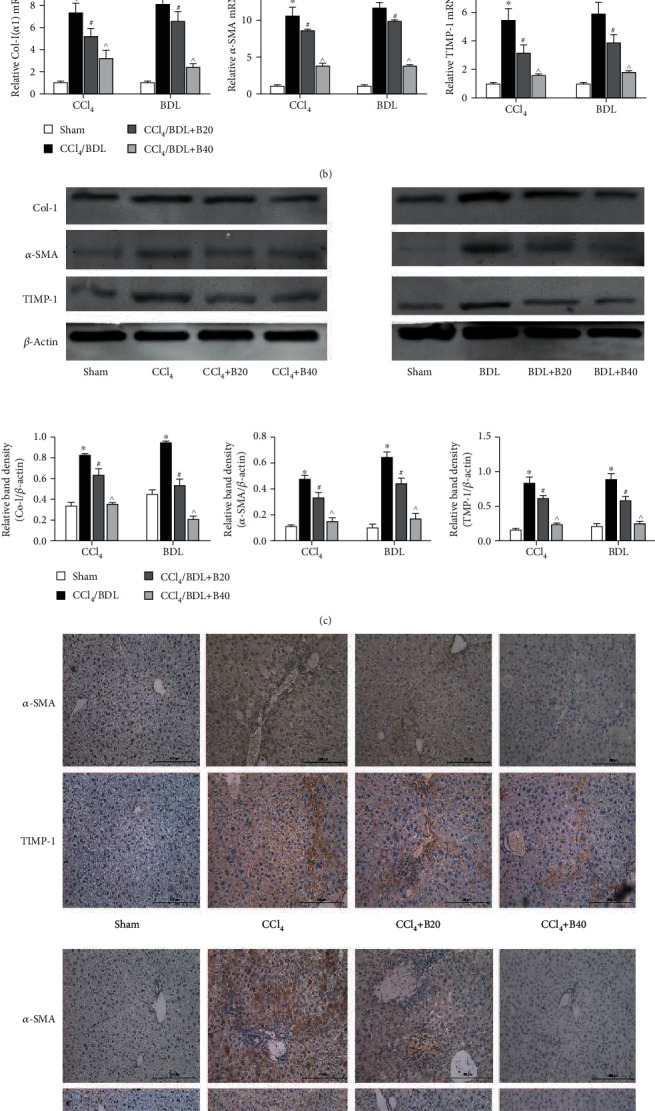
Bergenin inhibits the formation of the extracellular matrix. (a) Levels of serum HA and LN expressed as the mean ± SD (*n* = 8). (b) mRNA expression of collagen I, *α*-SMA, and TIMP1 assessed by real-time PCR (*n* = 8). (c) Protein expression of collagen I, *α*-SMA, and TIMP1 assessed by western blotting. The quantitative evaluation was determined by relative band density. (d) Immunohistochemical staining of *α*-SMA and TIMP1 (original magnification = 200x). ^∗^*p* < 0.05 for CCl_4_/BDL vs. sham, ^#^*p* < 0.05 for CCl_4_/BDL+B20 vs. CCl_4_/BDL, and ^^^*p* < 0.05 for CCl_4_/BDL+B40 vs. CCl_4_/BDL+B20.

The main components of ECM are hyaluronic acid (HA), fibronectin (FN), laminin (LN), type I collagen (CoI-I), *α*-SMA, matrix metalloproteinases (MMPs), and tissue inhibitors of metalloproteinases (TIMPs). The results showed that levels of HA and LN in the disease model groups were significantly increased, while those in the bergenin groups were significantly decreased, and the effect was proportional to the drug concentration (Figure 2(a)). Furthermore, we used molecular biological methods to measure levels of *α*-SMA, CoI-I, and TIMP1. At the transcriptional level, all were significantly increased in the CCl_4_ and BDL model groups, while high concentrations of bergenin downregulated the expression of these markers (Figures 2(b) and 2(c)). Similarly, the results of immunohistochemistry were consistent with the observed serum levels (Figure 2(d)). These results suggest that bergenin inhibits ECM depositions and thereby prevents liver fibrosis.

### 3.3. Bergenin Decreases Autophagy by Downregulating Beclin-1 and LC-3

**Figure 3:**
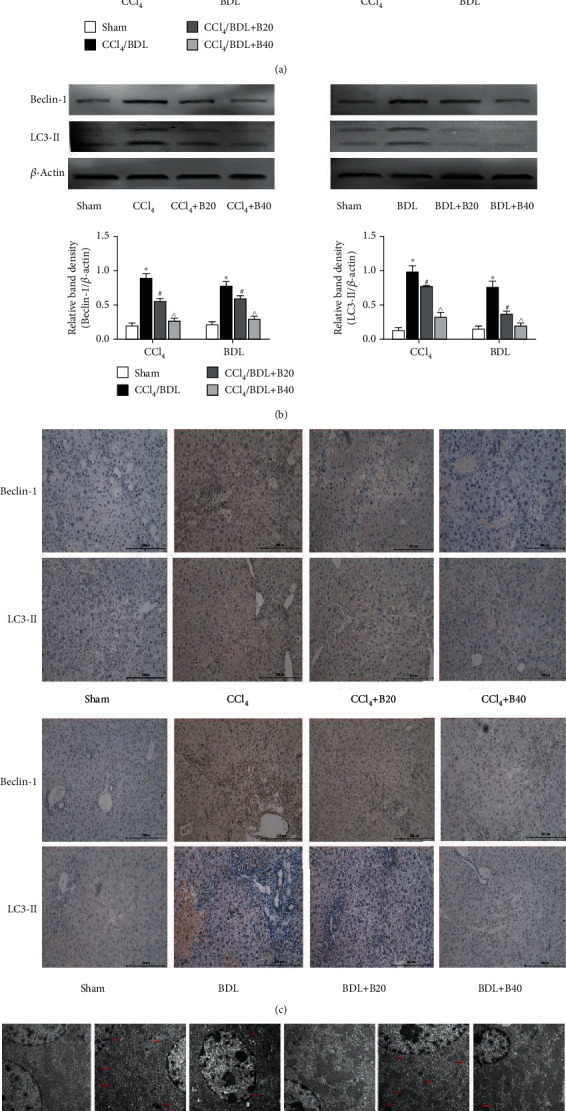
Bergenin decreases autophagy by downregulating Beclin-1 and LC-3. (a) mRNA expression of Beclin-1 and LC3-II assessed by real-time PCR (*n* = 8). (b) Protein expression of Beclin-1 and LC3-II assessed by western blotting. The quantitative evaluation was determined by relative band density. (c) Immunohistochemical staining of Beclin-1 and LC3-II (original magnification = 200x). (d) The amount of autophagosome significantly decreased as showed by TEM (original magnification: ×10000). ^∗^*p* < 0.05 for CCl_4_/BDL vs. sham, ^#^*p* < 0.05 for CCl_4_/BDL+B20 vs. CCl_4_/BDL, and ^^^*p* < 0.05 for CCl_4_/BDL+B40 vs. CCl_4_/BDL+B20.

Autophagy involves the phagocytosis of cytoplasmic proteins and organelles, their inclusion into vesicles, and fusion with lysosomes to form autophagic lysosomes, which provides energy for the activation of HSCs. The main proteins involved are LC-3II, Beclin-1, and p62. The results showed that mRNA levels of autophagy-related genes LC-3II and Beclin-1 in liver fibrosis model groups were significantly increased, while those in bergenin treatment groups were decreased in a dose-dependent manner (Figure 3(a)). In addition, western blotting and immunohistochemistry were used to measure the expression levels of tissue proteins, and the results were consistent with the gene transcription levels (Figure 3(b) and 3(c)). In the model group, more Beclin-1 and LC3-II proteins were stained with brown yellow particles by DAB compared with the sham group, while the positive area of the drug treatment group decreased with the increase of concentration. Electron microscopy was performed to observe autophagy directly, and the results showed that the number of autophagosomes was increased significantly in the disease model groups, but not in the drug groups (Figure 3(d)). In summary, bergenin could effectively reduce the levels of autophagy and block the energy supply needed for HSC activation.

### 3.4. Bergenin Inhibits the TGF-*β*1/Smads Pathway by Activating PPAR-*γ*

**Figure 4:**
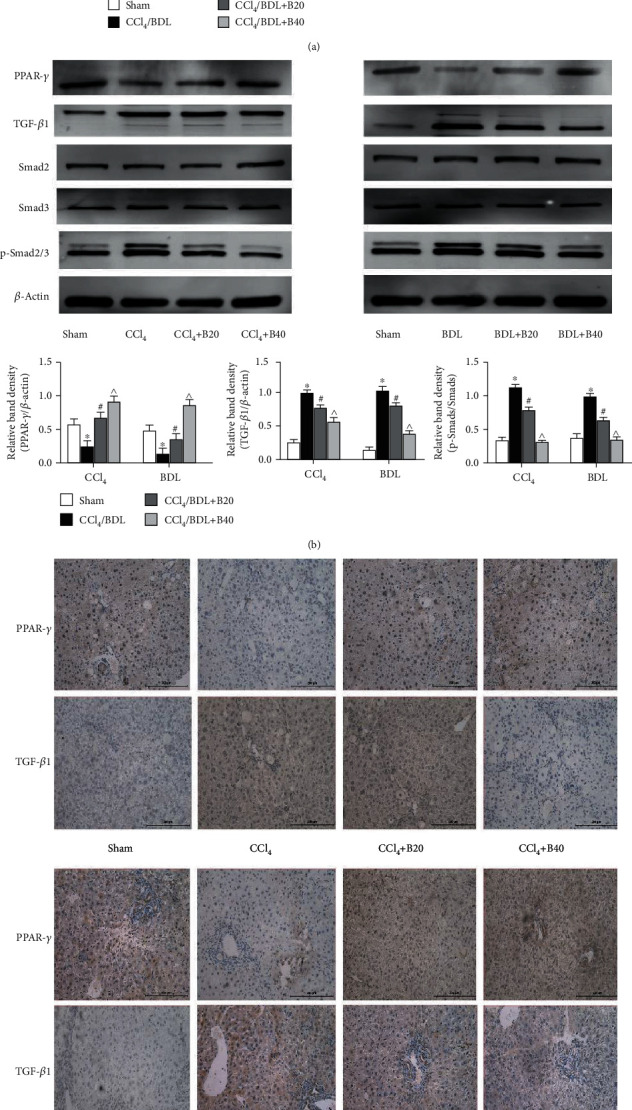
Bergenin inhibits the TGF-*β*1/Smads pathway by activating PPAR-*γ*. (a) mRNA expression of PPAR-*γ* and TGF-*β*1 assessed by real-time PCR (*n* = 8). (b) Protein expression of PPAR-*γ* and TGF-*β*1 assessed by western blotting. The quantitative evaluation was determined by relative band density. (c) Immunohistochemical staining of PPAR-*γ* and TGF-*β*1 (original magnification = 200x). ^∗^*p* < 0.05 for CCl_4_/BDL vs. sham, ^#^*p* < 0.05 for CCl_4_/BDL+B20 vs. CCl_4_/BDL, and ^^^*p* < 0.05 for CCl_4_/BDL+B40 vs. CCl_4_/BDL+B20.

TGF-*β*1 mediates necrosis and autophagy by activating phosphorylated Smads in the nuclear region. PPAR-*γ* is a key molecule regulating TGF-*β*1, which is known to be inhibited by bergenin. In order to clarify the mechanism of action of the drug, we analysed gene and protein expression levels of related pathways. The results showed that expression of PPAR-*γ* was decreased in the liver fibrosis model, but the expression was stimulated by the drug. However, TGF-*β*1, active Smad2, and Smad3 displayed the opposite trend (Figures 4(a) and 4(b)). Based on the consistent expression of total Smad2 and Smad3, levels of TGF-*β*1 and phosphorylated Smad2 and Smad3 were upregulated in the liver fibrosis model, but decreased in the drug group, and the differences were statistically significant. Immunohistochemical staining was also used to elucidate the changes in the expression of pathway molecules. In the model group, the brown granules in the nucleus of PPAR-*γ* were significantly decreased but were upregulated in the drug treatment group. On the contrary, more TGF-*β*1 proteins were stained by DAB into brown granules compared with the sham group, while the yellow area of the drug treatment group showed a downward trend (Figure 4(c)). These results suggest that bergenin can inhibit the TGF-*β*1/Smads pathway by activating PPAR-*γ*, thereby halting the progression of autophagy in liver fibrosis.

## 4. Discussion

Modern medicine has shown that liver fibrosis is a dynamic process. However, whether this process is progressive or related to the presence of liver injury factors and whether liver injury lesions continue to develop remain unknown. Research on liver fibrosis has recently entered a new era. Great progress has been made in understanding liver fibrosis and drug-targeted therapy [1, 25]. Natural plant extracts have been shown to exert strong biological effects, and bergenin is effective for decreasing fibrosis in various organs. Herein, we explored the protective effects of bergenin on the liver.

CCl_4_ is one of the most widely used chemical toxicants to induce liver fibrosis and cirrhosis in experimental animals. In the endoplasmic reticulum of hepatocytes, free radicals produced by CCl_4_ can bind covalently to macromolecules in hepatocytes after being activated by cytochrome P450 oxidase in liver microsomes, which leads to the production of reactive oxygen species and lipid peroxidation, resulting in liver fibrosis [26]. By contrast, the BDL model is characterised by cholestasis and inflammation due to blockage of the extrahepatic biliary system, leading to a strong fibrosis reaction around the portal vein. The two models complement each other and are used to comprehensively evaluate the effects of drugs.

First, we explored the effects of the drug on liver function and quantitatively evaluated liver function and the degree of liver fibrosis based on ALT, AST, and hydroxyproline. The results showed that liver enzymes and hydroxyproline were increased in the serum of the liver fibrosis model, while the drug effectively reduced the levels of these indicators, suggesting that bergenin could effectively inhibit the release of ALT and AST and reduce the production of collagen.

Pathological staining of hepatocyte necrosis and Masson staining of collagen formation directly reflected the effectiveness of the drug, consistent with previous studies [13]. Furthermore, activation of HSCs, phenotypic changes, and ECM deposition are the central links in the occurrence of liver fibrosis [1]. Therefore, it is of great significance to study ECM components when evaluating the severity of liver fibrosis. Our results showed that bergenin decreased levels of *α*-SMA, CoI-I, MMP2, and TIMP1. Therefore, bergenin may protect hepatocytes from hepatic fibrosis and inhibit the formation of key components of ECM in serum and tissues, thereby inhibiting the process of liver fibrosis.

Autophagy involves phagocytosis of cytoplasmic proteins and organelles, their inclusion into vesicles, and fusion with lysosomes to form autophagic lysosomes, which degrade their contents [27, 28]. Studies have shown that when HSCs are activated, autophagy increases to provide energy to promote the secretion of ECM components [29]. One study demonstrated that miR-96-5p inhibits the activation of HSCs by regulating ATG7 to block autophagy [30], and another demonstrated that fucoidan can inhibit ECM deposition and autophagy in liver fibrosis [15]. Therefore, if autophagy can be effectively inhibited, liver fibrosis may be inhibited to some extent. Beclin-1 and LC3-II are markers of autophagy that are increased significantly during liver fibrosis, and bergenin effectively reduced their levels in the present work. By contrast, expression of P62 was higher when autophagy was decreased and lower when autophagy was increased. Therefore, bergenin increased the expression level of P62 in tissues. These results are consistent with previous findings showing that inhibition of autophagy can significantly inhibit liver fibrosis.

PPAR-*γ* is a member of the nuclear transcription factor superfamily and can form a heterodimer with retinol X receptor (RXR) to regulate the expression of related genes [31, 32]. The TGF-*β*/Smads pathway is important in the process of liver fibrosis and can be regulated by PPAR-*γ*. Various drugs can inhibit TGF-*β* production by activating PPAR-*γ* during fibrosis in different tissues [33, 34]. PPAR-*γ* binds directly to Smad3 and inhibits the expression of connective tissue growth factor (CTGF) induced by TGF-*β* in smooth muscle cells [35].

**Figure 5:**
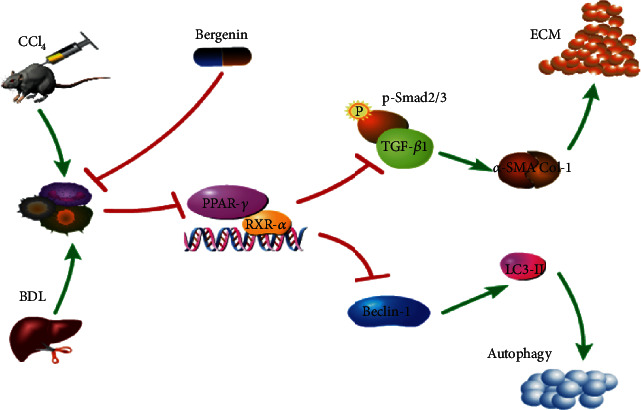
Mechanism of action of bergenin in liver fibrosis. The expression of PPAR-*γ* and RXR-*α* was increased, and the TGF-*β* pathway was inhibited following treatment with bergenin. The Smad2/3 that act downstream were not activated by phosphorylation and transported into the nucleus. Furthermore, Beclin-1 could not be bound to promote transcription, which reduced the likelihood of LC3-I to LC3-II transformation, which decreased fibrosis due to a lack of energy supply for HSC activation.

We also measured the expression of PPAR-*γ* and members of the TGF-*β*/Smads pathway in both disease model and drug treatment groups. The results showed that expression of PPAR-*γ* and RXR was increased to varying degrees following treatment with bergenin, which indicates that it might be a potential activator of PPAR-*γ*, similar to rosiglitazone and 15d-PGJ2 [36–39]. In the drug treatment group, the TGF-*β* pathway was inhibited, and Smad2/3 that act downstream were not activated by phosphorylation and transported into the nucleus. Furthermore, the specific DNA sequence of Beclin-1 could not be bound to promote transcription, which reduced the likelihood of LC3-I to LC3-II transformation, resulting in P62 accumulation and blockage of autophagy, which decreased fibrosis due to a lack of energy supply for HSC activation (Figure 5).

In conclusion, bergenin inhibits autophagy and blocks the energy supply required for HSC activation, thereby decreasing ECM formation and hepatocyte damage, which may affect the PPAR-*γ*/TGF-*β*/Smads axis. These findings establish bergenin as a potentially promising drug for the treatment of liver fibrosis.

## Figures and Tables

**Table 1:** Nucleotide sequences of primers used for qRT-PCR.

Gene		Primer sequence (5′-3′)
LC3-II	Forward	GACCGCTGTAAGGAGGTGC
	Reverse	AGAAGCCGAAGGTTTCTTGGG

Beclin-1	Forward	ATGGAGGGGTCTAAGGCGTC
	Reverse	TGGGCTGTGGTAAGTAATGGA

TIMP-1	Forward	CGAGACCACCTTATACCAGCG
	Reverse	ATGACTGGGGTGTAGGCGTA

*α*-SMA	Forward	CCCAGACATCAGGGAGTAATGG
	Reverse	TCTATCGGATACTTCAGCGTCA

TGF-*β*1	Forward	CCCCTGCAAGACCATCGAC
	Reverse	CTGGCGAGCCTTAGTTTGGAC

CoI-1*α*1	Forward	CAATGGCACGGCTGTGTGCG
	Reverse	AGCACTCGCCCTCCCGTCTT

PPAR-*γ*	Forward	GGAAGACCACTGCATTCCTT
	Reverse	GTAATCAGCAACCATTGGGTCA

*β*-Actin	Forward	CTGGAACGGTGAAGGTGACA
	Reverse	AAGGGACTTCCTGTAACAATGCA

## Data Availability

All data can be found in the study.
